# Sa12b Peptide from Solitary Wasp Inhibits ASIC Currents in Rat Dorsal Root Ganglion Neurons

**DOI:** 10.3390/toxins11100585

**Published:** 2019-10-10

**Authors:** Carmen Hernández, Katsuhiro Konno, Emilio Salceda, Rosario Vega, André Junqueira Zaharenko, Enrique Soto

**Affiliations:** 1 Instituto de Fisiología, Benemérita Universidad Autónoma de Puebla, Puebla 72570, Mexico; mary_car123@hotmail.com (C.H.); emilio.salceda@gmail.com (E.S.); axolotl_56@yahoo.com.mx (R.V.); 2 Institute of Natural Medicine, University of Toyama, Toyama 930-0194, Japan; kkgon@inm.u-toyama.ac.jp; 3 Laboratório de Genética, Instituto Butantan, São Paulo 05503-900, Brazil; a.j.zaharenko@gmail.com

**Keywords:** venom peptides, FMRF-amide, insect neurotoxin, protons, pH regulation, acid-sensing ion channels, acid-gated currents

## Abstract

In this work, we evaluate the effect of two peptides Sa12b (EDVDHVFLRF) and Sh5b (DVDHVFLRF-NH_2_) on Acid-Sensing Ion Channels (ASIC). These peptides were purified from the venom of solitary wasps *Sphex argentatus argentatus* and *Isodontia harmandi*, respectively. Voltage clamp recordings of ASIC currents were performed in whole cell configuration in primary culture of dorsal root ganglion (DRG) neurons from (P7-P10) CII Long-Evans rats. The peptides were applied by preincubation for 25 s (20 s in pH 7.4 solution and 5 s in pH 6.1 solution) or by co-application (5 s in pH 6.1 solution). Sa12b inhibits ASIC current with an IC_50_ of 81 nM, in a concentration-dependent manner when preincubation application was used. While Sh5b did not show consistent results having both excitatory and inhibitory effects on the maximum ASIC currents, its complex effect suggests that it presents a selective action on some ASIC subunits. Despite the similarity in their sequences, the action of these peptides differs significantly. Sa12b is the first discovered wasp peptide with a significant ASIC inhibitory effect.

## 1. Introduction

Acid-sensing ion channels (ASICs) are proton-gated Na^+^ channels of the ENaC/Degenerin channel family characterized by their sodium permeability, sensitivity to amiloride, and voltage insensitivity [1,2,3,4]. ASICs are widely distributed in the central and peripheral nervous systems, as well as in sensory and non-neuronal tissue [5]. Most functions of these channels have been described using inhibitors of ASIC channels combined with the use of knockout or knockdown animals [6]. The most potent and selective modulators of ASICs described to date are animal venoms obtained from spiders, snakes, and sea anemones [7,8,9].

FMRFa (Phe-Met-Arg-Phe amide) is an abundant tetrapeptide in invertebrate nervous systems, where it acts as a neurotransmitter and neuromodulator. RFa-related peptides share with FMRFa the characteristic C-terminus motive Arg-Phe-NH_2_ [10]. These neuropeptides are direct activators of two ion channels of the ENaC/Deg superfamily: the invertebrate FMRFa-gated Na^+^ channel (FaNaC) and the Hydra-RFa-gated Na^+^ channels (HyNaC) [11].

While FaNaC and HyNaC channels are activated by neuropeptides and modulated by acidic pH, ASICs are activated by pH and modulated by neuropeptides [12]. Several studies show that RFa-related peptides reduce desensitization and increase the sustained current and peak amplitude of ASIC currents [13,14,15,16,17,18]. These effects are pH-dependent, require the presence of the amide group, and are competitive with Ca^2+^. Three possible binding sites to ASIC have been proposed: the acidic pocket, the bottom of the thumb domain, and the central vestibule [19,20,21,22,23].

In this work, we studied the effects of two FMRFa related peptides (Sa12b and Sh5b [24,25] extracted from the venom of solitary wasps *Sphex argentatus argentatus* and *Isodontia harmandi*) on ASIC currents of rat dorsal root ganglion (DRG) neurons using the voltage clamp technique. We found that Sa12b exerts a potent inhibitory action on ASIC currents in DRG neurons.

## 2. Results

Stable proton-gated currents were recorded from 123 DRG neurons obtained from 32 rats (about 30% of the cells registered expressed a stable proton-gated current). These neurons had a membrane capacitance (C_m_) of 46.8 ± 1.45 pF (Gaussian fit shows a normal distribution of C_m_ r^2^ > 0.95), a resting membrane potential (V_m_) of −55.3 ± 1.4 mV, a membrane resistance (R_m_) of 137.5 ± 13.6 MΩ, an access resistance (R_a_) of 4 ± 0.3 MΩ and a membrane time constant (τ) of 131 ± 6.7 ms. Cell average diameter was 38.6 ± 6.8 μm, estimated from C_m_, which corresponds to medium-size DRG neurons according to Petruska’s classification [26].

### 2.1. ASIC Current in DRG Neurons

**Figure 1:**
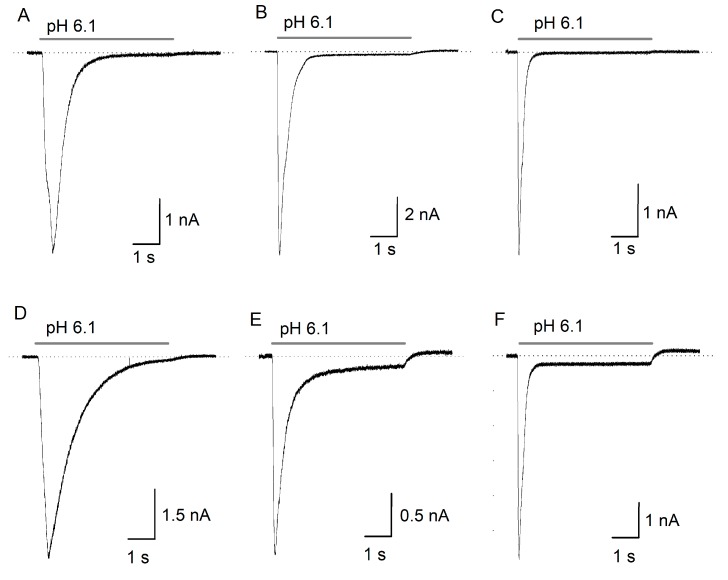
Diversity of acid-gated currents in dorsal root ganglion (DRG) neurons. The currents were elicited by a 5 s perfusion with a pH 6.1 solution. (**A**,**D**) depict currents with slow activation and slow desensitization (n = 9). (**B**,**E**), currents with rapid activation and intermediate desensitization (n = 28. C and F, currents with rapid activation and rapid desensitization (n = 58). (**A**–**C**) lack a sustained component (I_SS_), whereas (**D**–**F**) present it.

ASIC currents from isolated DRG neurons showed diverse characteristics, which result from the expression of ASIC heteromers and homomers of ASIC1–4 subunits in these neurons. The currents range from transient and rapid currents with partial or complete desensitization to currents with slow desensitization with a large sustained component (Figure 1). No clear groups could be formed to categorize the currents considering all the parameters analyzed, although according to their desensitization time constant (Ƭ_des_), we found that 61% of the registered currents showed Ƭ_des_ < 300 ms, 29.5% Ƭ_des_ > 300 and < 600 ms, while only the remaining 9.5% had slow kinetics with Ƭ_des_ > 600 ms.

Under control conditions, the pH gated currents activated at pH 6.1 showed a maximal inward peak current (I_peak_) of 4.5 ± 0.5 nA, a sustained component (I_SS_) of 0.09 ± 0.01 nA, a Ƭ_des_ of 348 ± 22 ms, and an integral of the current (I_int_) of 2.03 ± 0.171 nC. Current density had an average magnitude of 0.097 ± 0.009 nA/pF and a desensitization coefficient (I_SS_/I_peak_) of 0.04 ± 0.01 (mean ± ES, *n* = 95). None of the parameters obtained from the records show any correlation with the C_m_, which is an indicator of cell size.

### 2.2. Sa12b Action on ASIC Currents

**Figure 2:**
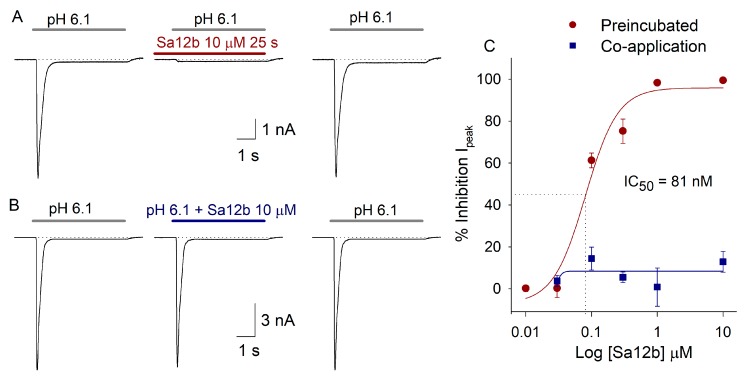
Effect of Sa12b on Acid-Sensing Ion Channels (ASIC) currents. In (**A**), recordings of ASIC current in dorsal root ganglion (DRG) neurons in control current, after 10 µM Sa12b preincubation application (20 s before and 5s during acid pulse) and after one recovery of 1 min washout. Sa12b inhibits the I_peak_ (98%) of ASIC current with 3% inhibition of I_SS_ component. In (**B**), 10 μM Sa12b co-application (peptide applied during 5 s along with acid pulse) inhibits I_peak_ 10% and increases I_SS_ by 18%. In (**C**), concentration-response relationship of Sa12b on ASIC I_peak_. Sa12b concentrations used were: 10, 30, 100, and 300 nM and 1 and 10 µM. The red circles represent the effect produced by preincubation, and the blue squares represent the effect during co-application of Sa12b (*n* = 34).

The peptide was initially tested at 10 μM, this concentration was used because it is close to the EC_50_ of FMRFa. With application of 10 µM Sa12b in preincubation form (20 s before and 5 s during acid pulse), Sa12b inhibited ASIC currents 92.7 ± 7.3% (*p* < 0.05 paired Student’s *t*-test, *n* = 5) (Figure 2A). In contrast, during co-application (toxin applied only during the 5 s of acid pulse) Sa12b produced a non-significant inhibition of ASIC currents of 12.9 ± 4.9% (*n* = 4) (Figure 2B).

**Figure 3:**
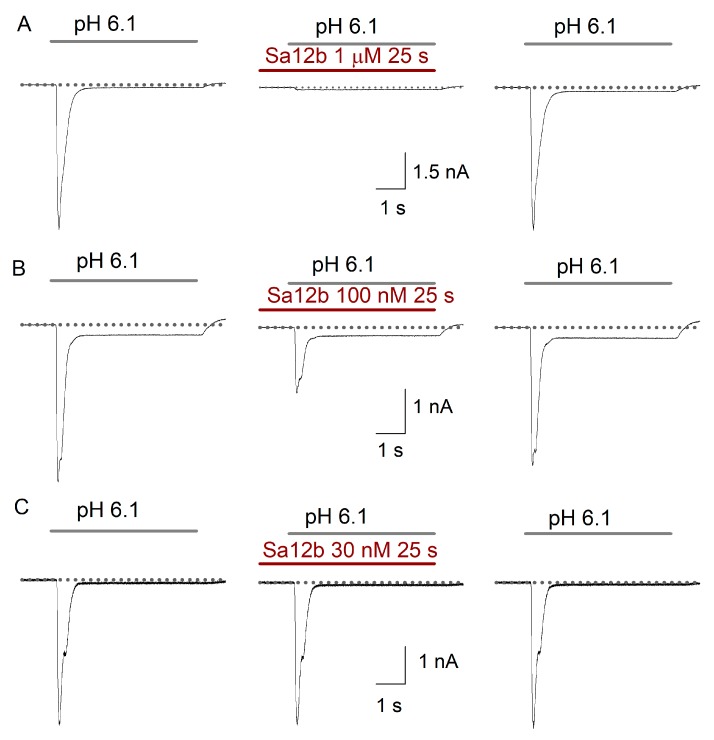
Typical traces of the effect of preincubation perfusion with Sa12b on the ASIC current at the different tested concentrations. In (**A**), the use of 1 µM Sa12b produced a reversible nearly total inhibition of the I_peak_ with no effect on the small I_SS_ component. In (**B**), 100 nM Sa12b caused an inhibition of I_peak_ and I_SS_ of 66% and 7%. In (**C**), 30 nM Sa12b produced an inhibition of I_peak_ and I_SS_ of 2% and 10%, respectively. The inhibitory effects of Sa12b were completely reversed after 1 min washout of the toxin. The dotted lines indicate the zero current, and the horizontal bars show Sa12b preincubation and pH 6.1 perfusion.

The inhibitory action of Sa12b in I_peak_ was dose-dependent with preincubation application (Figure 2A–C and Figure 3), it was adjusted with a dose-response function with an IC_50_ = 81 ± 29.4 nM, H = 1.8 and r^2^ = 0.97 (*n* = 34). All Sa12b effects were fully reversed after 1 min washout of the peptide. Other parameters of ASIC current were not significantly modified by Sa12b perfusion except for the Ƭ_des_, which increased 28% with 300 nM Sa12b (Table 1).

To determine whether or not the effect of Sa12b was selective for some type of ASIC current, a correlation analysis between the current properties (I_peak_, Ƭ_des_, and I_SS_) and the effect of Sa12b was performed. It was found that the inhibitory effect of Sa12b on ASIC currents does not depend on the C_m_, the Ƭ_des_, or the I_SS_/I_peak_ of the control currents; regardless of the concentration tested either during preincubation or co-application of the peptide.

### 2.3. pH Activation Versus Sa12b Effect

**Figure 4:**
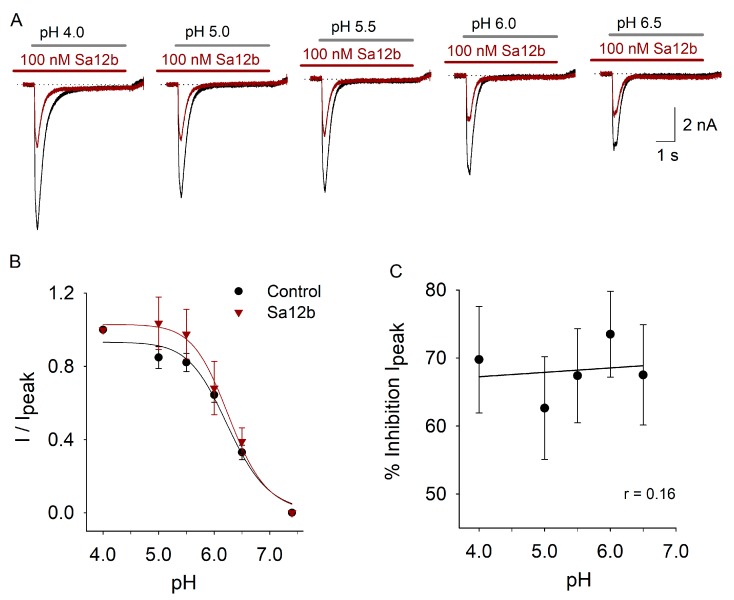
Effect of pH-gating the ASIC current on Sa12b action. In (**A**), recordings of ASIC current at different gating pHs in control (black traces) and after preincubation with 100 nM Sa12b (red traces). The inhibitory effect of Sa12b is similar regardless of the pH used to activate the current. All recordings are from the same cell, which desensitize nearly completely and shows small I_SS_ component. In (**B**), relation between the pH used to activate the current and the normalized I_peak_ in control (black) and after 100 nM Sa12b. Data were adjusted with a sigmoidal function, no significant difference between pH sensitivity of the current in control (pH_50_ = 6.26 ± 0.1) and with Sa12b (pH_50_ 6.27 ± 0.06) was found. The pHs used to activate the current were: pH 6.5, *n* = 15; pH 6.0, *n* = 15; pH 5.5, *n* = 16; pH 5.0, *n* = 17; pH 4.0, *n* = 14. In (**C**), plot of the percent I_peak_ inhibition produced by 100 nM Sa12b against pH. The data were adjusted with a linear function showing that inhibition produced by Sa12b is independent of the pH used to activate the ASIC current.

To determine whether or not Sa12b action is pH-dependent, the effect of Sa12b 100 nM (concentration close to IC_50_) was analyzed as a function of pH used to activate the current (pH from 4.0 to 6.5). As previously described, the current amplitude increased as a function of proton concentration (Figure 4A,B). The pH which activated 50% of the ASIC current (pH_50_) was about 6.1 as previously described for ASIC currents in DRG neurons [27]. The relationship between pH and proton-gated current amplitude in the presence of 100 nM Sa12b showed no significant difference with that found in control condition (Figure 4B). Analysis of percent inhibitory effect of 100 nM Sa12b as a function of pH showed that pH gating of the current did not significantly modify the inhibitory action of Sa12b (Figure 4C). These data indicate that Sa12b does not interact with the proton-gating mechanism of ASICs.

### 2.4. Effect of Sh5b

As with Sa12b peptide, the application of Sh5b peptide was carried out under co-application and preincubation. The concentrations at which the peptide was tested were: for co-application 100 nM, 3 μM, 10 μM, and 30 μM; for preincubation application, the concentrations were 100 nM, 1 and 10 μM.

**Figure 5:**
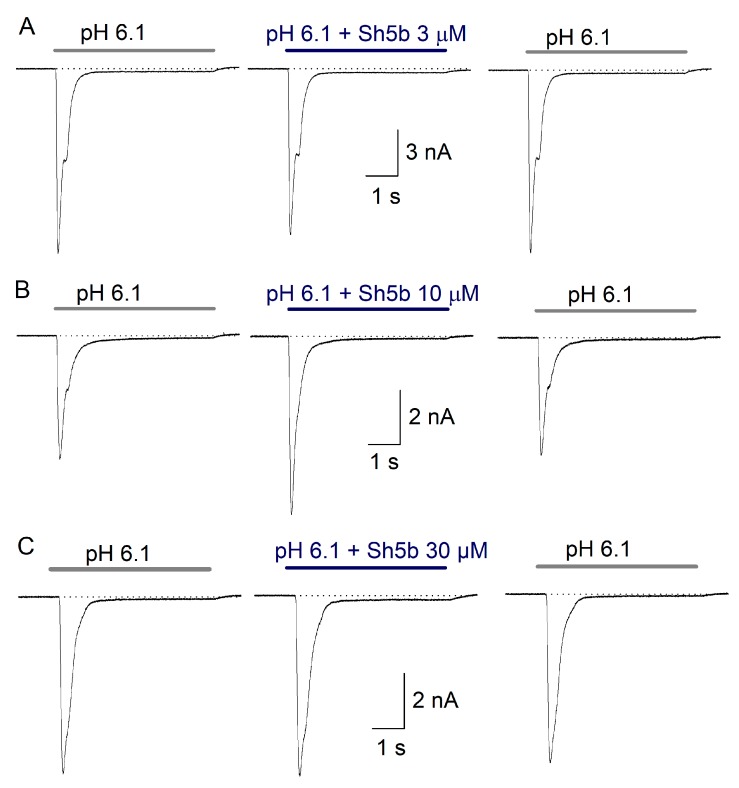
Effect of Sh5b peptide on ASIC currents of DRG neurons. Graphs show ASIC current in control, co-application, and after one minute of peptide washout. In (**A**), co-application of 3 µM Sh5b reduced I_peak_ of ASIC currents by 9.6%, while I_SS_ was increased by 30%. In (**B**), co-application of 10 µM Sh5b, increased I_peak_ by 44.6% and I_SS_ 14.6%. In (**C**), the co-application of 30 μM Sh5b caused an inhibition of 3.8% on I_peak_ and an increase of 102% on I_SS_ from 79 pA to 159 pA. Effects were reversed by 1 min peptide washout.

In the co-application protocol, 100 nM Sh5b (*n* = 5) showed no consistent concentration-dependent effects on the analyzed parameters. At 3 µM Sh5b (*n* = 12) decreased the Ƭ_des_ by 7% (*p* = 0.046) with highly variable non-significant increase of the I_SS_. Increasing Sh5b concentration to 10 μM produced an increase of the I_peak_ in some cells and a decrease in other group, but overall change was non-significant (Table 2). Other parameter changes were also non-significant. At 30 µM Sh5b (*n* = 6) increased the I_SS_ 78 ± 16% (*p* = 0.004) (Figure 5). However, the observed effects on ASIC current when using Sh5b in co-application were not dependent on concentration (Table 2).

**Figure 6:**
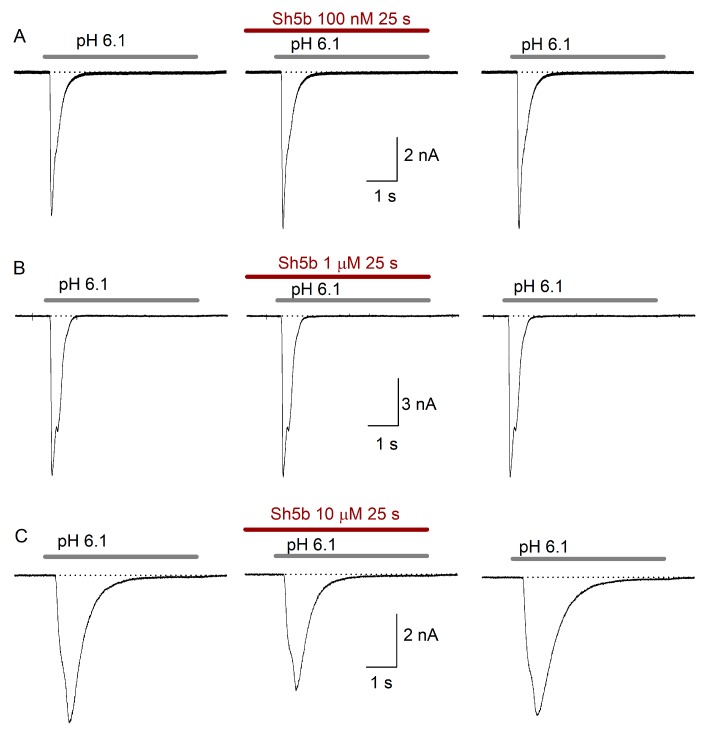
Graphs showing the effect of Sh5b preincubation on ASIC currents. Recordings show ASIC current under control conditions, after preincubation with Sh5b and after one-minute peptide washout. In (**A**), shows the effect of preincubation with 100 nM Sh5b, which produced a marginal increase of the I_peak_ and I_SS_ of 1% and 3% respectively, with no change in Ƭ_des_. In (**B**), preincubation with 1 µM Sh5b inhibits I_peak_ and I_SS_ by 1% and 5% respectivley. In (**C**), use of 10 µM Sh5b inhibits the I_peak_ by 18% and increased the I_SS_ by 26%, with no change in Ƭ_des_. The effect was reversed by 1 min washout of the peptide.

The use of 100 nM Sh5b in preincubation (*n* = 9) produced no effect on the analyzed parameters. Perfusion of 1 µM Sh5b (*n* = 5) produced a marginal decrease of the I_peak_ (9 ± 5%), and an increase of the I_SS_, both effects were non-significant. 10 µM Sh5b (*n* = 12) did not produced significant effects on the studied parameters either (Figure 6, Table 2).

## 3. Discussion

### 3.1. Sa12b

Sa12b peptide, when applied by preincubation, reversibly inhibits the amplitude of the peak of ASIC currents (IC_50_ ~ 81 nM) in rat DRG neurons in a concentration-dependent manner without consistent action on the time course of desensitization or the sustained component of the current. Currents activated by H^+^ in DRG neurons are heterogeneous due to the combination of two or more ASIC subunits with coexistence of multiple populations of channels in the same cell [28,29,30,31]. The inhibitory effect of Sa12b was similar in all cells regardless of the kinetics of currents, which indicates that Sa12b action is not specific to any particular ASIC subunit; however, this question requires further exploration on channels expressed in a heterologous system.

We found no effect on the I_peak_ during co-application of Sa12b and acidic pH. That the inhibitory effect of Sa12b was observed only after preincubation application suggests that the peptide needs to interact with the channel during its closed state; an alternative explanation would be that this effect is due to a slow interaction of the peptide with ASIC or a slow conformational change of the channel induced by Sa12b [21]. RFa-related peptides also seem to produce their modulating effect only when applied before acid gating of the channel [20].

The inhibitory potency of Sa12b (IC_50_ = 81 nM) on ASIC currents is comparable to the inhibition caused by peptides of vegetable and animal origin, such as chlorogenic acid (CGA, polyphenol) and the gastrodin (phenol) that inhibits ASIC currents in rat DRG neurons (IC_50_ ~ 230 nM and ~ 210 nM respectively) [32,33]. APETx2 (from the sea anemone *Anthopleura elegantissima*) inhibits the homomeric channels of rASIC3 (IC_50_ 37–63 nM) and hASIC3 (IC_50_ ~ 175 nM) [34]; mambalgines (from the black mamba and the green mamba) inhibit the homomers of ASIC1a and ASIC1b, and the heteromers containing ASIC1a with an IC_50_ ranging from 11 to 250 nM [35,36]; or PhcrTx1 peptide extracted from *Phymanthus crucifer* (IC_50_ ~ 100 nM) which inhibits ASIC currents ≅ 40% [37]. The inhibitory effect of Sa12b in ASIC currents is only surpassed in potency by two known ASIC1a inhibitors: PcTx1, from tarantula venom *Psalmopoeus cambridgei* (IC_50_ = 1 nM) [38] and Hi1a, from the venom of the Australian spider *Hadronyches infensa* (IC_50_ = 0.4–0.5 nM), however, PcTx1 behaves as an agonist of ASIC1b (EC_50_ ~ 100 nM), while Hi1a produces an incomplete current inhibition at saturating concentration (1 μM) [9]. It is worth note that Sa12b produces a close to 100% inhibition of I_peak_ at 1 µM. Which suggests that Sa12b exerts an unspecific action among ASIC subunits, although the lack of inhibition of I_SS_ suggest some kind of selectivity among ASIC subunits. To define this, it will be needed to perform experiments in a heterologous expression system studying the action of Sa12b on specific homomers of ASIC subunits.

It is speculated that modulation of ASIC channels by RFa-related peptides is due to direct interaction between the peptide and the extracellular domain of the channel having the lower region of the palmar domain of the channel as a probable binding site, specifically the region occupied by the central vestibule of the channel; furthermore, it has been suggested that RFa-related peptides bind to the channel in the closed state and dissociate very slowly from the desensitized state [22]. Since Sa12b has a very short amino acid sequence, binding in the central vestibule may plug the channel, decreasing the conductance instead of slowing inactivation and desensitization of ASIC currents, which is what RFa does.

Sa12b sequence (EDVDHVFLRF) suggests the presence of a hydrophobic patch provided by the amino acids: Val3, Val6, Phe7, Leu8, and Phe10; the Phe15 residue in APETx2 is of great importance for this toxin to inhibit the currents of ASIC3 [39]. Similarly, PcTx1 has a hydrophobic patch conferred by Trp7 and Trp24 which interacts with the thumb domain of the ASIC channel, while the basic group of PcTx1 (Arg26, Arg27, and Arg28) enters the acidic pocket to form strong hydrogen bonds [40]. Sa12b also possesses two residues with a positive charge (His5 and Arg9) that could be a binding site with ASIC channels.

### 3.2. Sh5b

Sh5b did not produce consistent, reproducible, effects on ASIC currents, it shows various effects on most of the analyzed parameters, including dual effects on the I_peak_ and Ƭ_des_. The complex action of Sh5b suggests that this peptide presents selective action on some subunits of ASICs. As already mentioned, the macroscopic currents activated by H^+^ in the DRG neurons present a morphological heterogeneity resulting from the combination of two or more ASIC subunits, so the inconsistent action of Sh5b could be given by selectivity of the peptide for some ASIC subunits. Future studies using heterologous expression of ASIC subunits could clarify whether Sh5b possesses any selectivity; if so, Sh5b can become a pharmacological tool that allows studying specific ASIC subunits.

Application of Sh5b by preincubation showed a tendency to inhibit the current peak. The I_SS_ component increased slightly after application of Sh5b, and the I_SS_/I_peak_ relationship also increased; this last parameter was the one that had statistically significant effects in the greatest number of the tested concentrations, which suggests a modification on the desensitization process of ASICs. However, the Ƭ_des_ and the integral of the current show no consistent changes, exhibiting dual effects. During co-application, the I_SS_/I_peak_ relationship showed a tendency towards the increase, but the Ƭ_des_ did not show noticeable differences.

RFa-related peptides have an NH_2_ group which is positively charged at pH 5 to 8 [21]. The effects of Sh5b could be due to the positive charge given by its amine group. Other inhibitors of ASICs, such as the PhcrTx1 peptide, which at pH 7.4 has a net charge of +5.03, APETx2 (net charge = +2.00), and PcTx1 (net charge = +3) [37] also have that particularity. Aminoglycosides are also positively-charged ASICs modulators [27].

Analysis of the structure of Sa12b (EDVDHVFLRF) and Sh5b (DVDHVFLRFa) showed that Sa12b has an extra Glu in its N-terminal, while Sh5b has an amide residue in its C-terminal. Sa12b has three negatively charged amino acids (Glu1, Asp2, and Asp4) and two positively charged ones (His5 and Arg9), which gives the peptide a negative net charge, besides it has four polar amino acids (Glu1, Asp2, Asp4, and Arg9) and six apolar ones (Val3, His5, Val6, Phe7, Leu8, Phe10). In contrast, Sh5b presents in its sequence two negatively charged amino acids (Asp1 and Asp3) and two positively charged ones (His4 and Arg8), which makes it a peptide with neutral charge. With respect to solubility, Sh5b consists of three polar amino acids (Asp1, Asp3, and Arg8) and six apolar ones (Val2, His4, Val5, Phe6, Leu7, Phe9). These structural differences, mainly the difference of net charges, could favor differences in the folding of the tertiary structure, which could produce the differences in the interaction of Sa12b and Sh5b with ASIC.

## 4. Conclusions

The results from this work show that the application of Sa12b exerts an inhibitory effect on ASIC currents from DRG neurons, this effect was concentration-dependent and reversed after washout of the peptide. Since the inhibition was close to 100% at 1 μM and all ASIC subunits are expressed in DRGs, it suggests that Sa12b inhibits different ASIC subunits without an apparent selectivity. Sa12b is the first discovered wasp peptide with a significant ASIC inhibitory action.

## 5. Material and Methods

### 5.1. Animals and Cell Culture

To study the effect of Sa12b and Sh5b on ASIC, DRG neurons were obtained from Long-Evans CII / ZV rats of 7 to 10 days of postnatal age, of either sex. Animals were provided by the laboratory animal facility ‘Claude Bernard’ of the Autonomous University of Puebla. The study was performed in accordance with the recommendations in the Guiding Principles in the Care and Use of Vertebrate Animals in Research and Training of the American Physiological Society, and with the regulations of the NOM-062-ZOO-1999 of the Mexican Ministry of Agriculture, Stockbreeding, Rural Development, Fishing and Food. The protocol was reviewed and approved by the Institutional Committee for Animal Care and Use (IACUC) of the Autonomous University of Puebla (VIEP-BUAP) on 17 July 2017. The ethical approval code is SOEE-UALVIEP-17-1. All efforts were made to minimize animal suffering and to reduce the number of animals used. DRG neurons were isolated and maintained in primary culture according to the methodology described previously [41]. The dissection and cell culture were performed within a level I biosafety laminar flow hood (Nuaire, Plymouth, MN, USA). Rats were anesthetized with sevoflurane and sacrificed by decapitation. Subsequently, the rat was placed in prone position to make a longitudinal incision through the vertebral bodies removing the spinal cord. Dorsal root ganglia were isolated (approximately 12 to 18 per rat) using conventional dissection under a stereoscopic microscope (American Optical, Southbridge, MA, USA). Once extracted, DRG neurons were placed in a disposable sterile centrifuge tube (Corning, Corning, NY, USA), in which they were incubated for 30 min at 37 °C in Leibovitz L15 medium (L15) (Invitrogen, Waltham, MA, USA), added with 1.25 mg/mL of trypsin and 1.25 mg/mL of collagenase (both from Sigma-Aldrich, St. Louis, MO, USA) for an enzymatic dissociation.

After the enzymatic treatment, the ganglia were washed 3 times with 100% L15 medium; after each wash, the cells were subjected to mechanical dissociation using glass three-gauge Pasteur pipettes; between each wash, a cell pellet was formed using a centrifuge at 5000 rpm. After the third wash, once the cell pellet was formed, the supernatant was discarded, and the cell suspension was placed in a 35 mm culture dish (Corning) on 12 × 10 mm glass plates (Corning) previously treated with poly-D-lysine (Sigma-Aldrich, St. Louis, MO, USA).

Dissociated neurons were incubated for a period of time ranging from 2 to 8 h in a humidified atmosphere (95% O_2_, 5% CO_2_, at 37 °C) using a water-jacketed CO_2_ incubator (Nuaire, Plymouth, MN, USA) allowing for settlement and adhesion of isolated cells to the glass plates. The cells were cultured in modified L15 medium, supplemented with 10% fetal bovine serum (Gibco, Waltham, MA, USA), 100 IU penicillin (Lakeside, Hayward, CA USA), fungizone 2.5 μL/mL (Gibco), NaHCO_3_ 15.7 mM (J.T. Baker, Radnor DE, USA) and 15.8 mM HEPES (Sigma-Aldrich, St. Louis, MO, USA).

### 5.2. Recording of ASIC Currents in DRG Neurons

After an incubation period of 2 to 8 h, the recording of ASIC currents in DRG neurons was performed. Cells were transferred to a recording chamber mounted in a phase-contrast inverted microscope (TMS, Nikon Co. Tokyo, Japan). Neurons that were not attached to other cells, and that had a round or ovoid shape (without dendritic or axonal extensions) with a delimited refringent membrane were chosen for recording.

The recording chamber was constantly perfused with extracellular solution (Table 3). Recordings were performed in whole-cell voltage-clamp mode using an Axopatch 1D amplifier (Molecular Devices, Union City, CA, USA). Data collection and generation of commands for the perfusion change were carried out by the pClamp 9.2 (Molecular Devices) software in a 16-bit data acquisition system (Digidata 1320, Molecular Devices). Microelectrodes were made from borosilicate glass capillaries (TW120-3; WPI, Sarasota, FL, USA) with a micropipette puller (80-PC; Sutter Instruments Company, San Rafael, CA, USA), which once filled with the intracellular solution (Table 3) had a resistance of 1.4 to 3.1 MΩ. The signals were digitized at 5 kHz. The series resistance was electronically compensated at 80%. Throughout the recording, access resistance and seal quality were monitored to ensure stable recording conditions. The records that showed a > 10% change in access resistance compared to the initial conditions were excluded from data analysis.

Proton-gated currents were obtained with a holding potential of −60 mV. Cells were subjected to a test protocol with an acid solution of pH 6.1 for 5 s (Table 3). In all the experiments at least two control recordings were made before performing some type of experimental manipulation in order to guarantee that the cells expressed a stable proton-gated current; the margin of variation in the amplitude of the current between one control recording and another should be less than 10%.

### 5.3. Wasp Peptides

The peptides Sa12b (EDVDHVFLRF, molecular weight = 1276.4 g/mol) and Sh5b (DVDHVFLRF-NH2, molecular weight = 1146.3 g/mol) were purified from the venom extracts of solitary wasps *Sphex argentatus argentatus* and *Isodontia harmandi*, respectively, and the structure was determined by MALDI-TOF/TOF MS analysis (manuscript in preparation). The synthetic specimens of these peptides were used in this study.

### 5.4. Peptide Synthesis

The peptide was synthesized on an automated PSSM-8 peptide synthesizer (Shimadzu Corp., Kyoto, Japan) by a stepwise solid-phase method using N-9-fluorenylmethoxycarbonyl (Fmoc) chemistry. All the resins and Fmoc-L-amino acids were purchased from HiPep Laboratories (Kyoto, Japan). Cleavage of the peptide from the resin was achieved by treatment with a mixture of TFA/H_2_O/triisopropylsilane (TIS) (95:2.5:2.5) at room temperature for 2 h. After removal of the resin by filtration and washing twice with trifluoroacetic acid (TFA), the combined filtrate was added dropwise to diethyl ether at 0 °C and then centrifuged at 3000 rpm for 10 min. Thus, obtained crude synthetic peptide was purified by semipreparative reverse-phase HPLC using CAPCELL PAK C_18_, 10 × 250 mm with isocratic elution of 20–25% CH_3_CN/H_2_O/0.1% TFA at a flow rate of 3 mL/min. The homogeneity and the sequence were confirmed by MALDI-TOF MS and analytical HPLC.

### 5.5. Experimental Design and Data Analysis

ASIC currents were activated by micro-perfusion of the cell under recording with an acid solution (pH 6.1) through a square tube using a rapid perfusion exchange system (SF-77B, Warner Inst., Hamden, CT, USA). The pH-gated current was activated using a pH of 6.1 which coincides with the pH_50_ previously demonstrated for ASIC currents in DRG neurons [41]. Capsazepine 10 μM was added to the extracellular solution at pH 6.1 in order to limit the activation of TRPV1 receptors, which are also sensitive to acid and are expressed in DRG neurons [42]. To study the effect of Sa12b and Sh5b peptides on ASIC currents of DRG neurons two application protocols were used [41]. Peptides were applied by sustained application (preincubation) and by co-application. In the preincubation, the toxin was applied through the pH 7.4 extracellular solution for 20 s before the acid pulse and during the 5 s that the acid pulse lasted. In co-application, the compound was applied only during the 5-second acid pulse. The effects observed during preincubation result from channel exposure to the peptide during the closed, open, and desensitized states. During co-application the toxins interacts with the channel in the open and desensitized states.

The passive properties of the neurons were recorded in each experiment, including the membrane capacitance (C_m_), cell-membrane voltage (V_m_), membrane resistance (R_m_), and access resistance (R_a_). Solutions were prepared at the time of experiment; the peptides were kept frozen at −20 °C in aliquots at different concentrations in deionized water added with 1 mg/mL of albumin (Sigma-Aldrich) to prevent the peptides from adhering to the walls of the perfusion tubes.

The proton-gated currents were processed offline using the software Clampfit 9.2 (Molecular Devices), Microsoft Office Excel 2010 and SigmaPlot 12.0. For each experimental condition two control recordings were obtained, one recording with the application of the peptide, and two washout recordings. The problem currents were normalized with respect to the average of the control currents in order to obtain the percentage of change in the parameters measured in the presence of the toxins.

For the analysis of the toxin actions, the concentration-response curves were adjusted with a Hill equation:

Y = A_2_ + (A_1_ − A_2_)/(1 + (x/E_50_) H)

where: Y = Pharmacological effect, x = Concentration tested, A_1_ and A_2_ = Maximum and minimum effect, E_50_ = Concentration in which 50% of the effect is obtained, H = Hill constant.

To study the desensitization of the current, a simple exponential function was adjusted, obtaining the decay constant of the current (Ƭ_des_).

To determine the statistical significance of the data, a paired Student’s *t*-test was used and *p* values reported; the experimental data are presented as the mean ± E.S.

## Figures and Tables

**Table 1:** Effect of Sa12b on macroscopic Acid-Sensing Ion Channels (ASIC) currents in dorsal root ganglion (DRG) neurons.

	[Sa12b]	*n*	% I_peak_	% I_SS_	% Ƭ_des_
Co-application	30 nM	5	🡹 4 ± 3	🡹 39 ± 24 (*p* = 0.02)	🡻 4 ± 3
	100 nM	4	🡻 14 ± 5	🡻 21 ± 16	🡹 5 ± 1
	300 nM	6	🡻 5 ± 3	🡻30 ± 14	🡹 3 ± 5
	1 µM	5	🡹 1 ± 9	🡹 15 ± 14	🡹 4 ± 4
	10 µM	4	🡻 13 ± 5	🡻 29 ± 19	🡻 2 ± 5
Preincubation Application	30 nM	6	🡻 0.2 ± 4	🡹 19 ± 22	🡹 0.03 ± 4
	100 nM	5	🡻 63 ± 4 (*p* = 0.003)	🡹 4 ± 21	🡹 17 ± 18
	300 nM	9	🡻 76 ± 5 (*p* = 0.0002)	🡹 81 ± 51	🡹 28 ± 12 (*p* = 0.048)
	1 µM	5	🡻 98 ± 1 (*p* = 0.03)	🡻 13 ± 27	
	10 µM	5	🡻 92 ± 7 (*p* = 0.04)	🡻 31 ± 20	

The effects that presented a significant difference are shown in red. The upward arrows indicate an increase and the downward arrows indicate a decrease. Student´s *t*-test.

**Table 2:** Effect of Sh5b on macroscopic ASIC currents in DRG neurons.

	[Sa12b]	n	% I_peak_	% I_SS_	% Ƭ_des_
Co-application	100 nM	5	🡻2 ± 4	🡹 85 ± 74	🡻 1 ± 2
	3 µM	12	🡻6 ± 3	🡹 116 ± 104	🡻 7 ± 4
	10 µM	9	🡹17 ± 15	🡹 1 ± 11	🡻 10 ± 5
	30 µM	6	🡻1 ± 6	🡹 78 ± 16	🡹 9 ± 5
Preincubation application	100 nM	9	🡻0.5 ± 6	🡹 53 ± 38	🡹 13 ± 15
	1 µM	5	🡻9 ± 5	🡹 14 ± 41	🡹 3 ± 5
	10 µM	12	🡻11 ± 4	🡻 8 ± 15	🡻 5 ± 4

The effects that presented a significant difference are shown in red. The upward arrows indicate an increase and the downward arrows indicate a decrease. Student´s *t*-test.

**Table 3:** Solutions used for electrophysiological recording.

	Extracellular [mM]	Acid Solution [mM]	Intracellular [mM]
NaCl	140	140	10
KCl	5.4	5.4	125
CaCl_2_	1.8	1.8	0.134
MgCl_2_	1.2	1.2	-
HEPES	10	-	5
MES	-	10	-
D-glucose	10	10	-
EGTA	-	-	10
ATP- Mg	-	-	2
GTP-Na	-	-	1
	adjusted to pH 7.4 with NaOH	adjusted to desired pH with NaOH	adjusted to pH 7.2 with KOH
